# Age-Related Macular Degeneration: New Paradigms for Treatment and Management of AMD

**DOI:** 10.1155/2018/8374647

**Published:** 2018-02-01

**Authors:** Luis Fernando Hernández-Zimbrón, Ruben Zamora-Alvarado, Lenin Ochoa-De la Paz, Raul Velez-Montoya, Edgar Zenteno, Rosario Gulias-Cañizo, Hugo Quiroz-Mercado, Roberto Gonzalez-Salinas

**Affiliations:** ^1^Research Department, Asociación Para Evitar la Ceguera, México City, Mexico; ^2^Biochemistry Department, School of Medicine, UNAM, 04510 México City, Mexico; ^3^Retina Department, Asociación Para Evitar la Ceguera, México City, Mexico

## Abstract

Age-related macular degeneration (AMD) is a well-characterized and extensively studied disease. It is currently considered the leading cause of visual disability among patients over 60 years. The hallmark of early AMD is the formation of drusen, pigmentary changes at the macula, and mild to moderate vision loss. There are two forms of AMD: the “dry” and the “wet” form that is less frequent but is responsible for 90% of acute blindness due to AMD. Risk factors have been associated with AMD progression, and they are taking relevance to understand how AMD develops: (1) advanced age and the exposition to environmental factors inducing high levels of oxidative stress damaging the macula and (2) this damage, which causes inflammation inducing a vicious cycle, altogether causing central vision loss. There is neither a cure nor treatment to prevent AMD. However, there are some treatments available for the wet form of AMD. This article will review some molecular and cellular mechanisms associated with the onset of AMD focusing on feasible treatments for each related factor in the development of this pathology such as vascular endothelial growth factor, oxidative stress, failure of the clearance of proteins and organelles, and glial cell dysfunction in AMD.

## 1. Introduction

**Figure 1:**
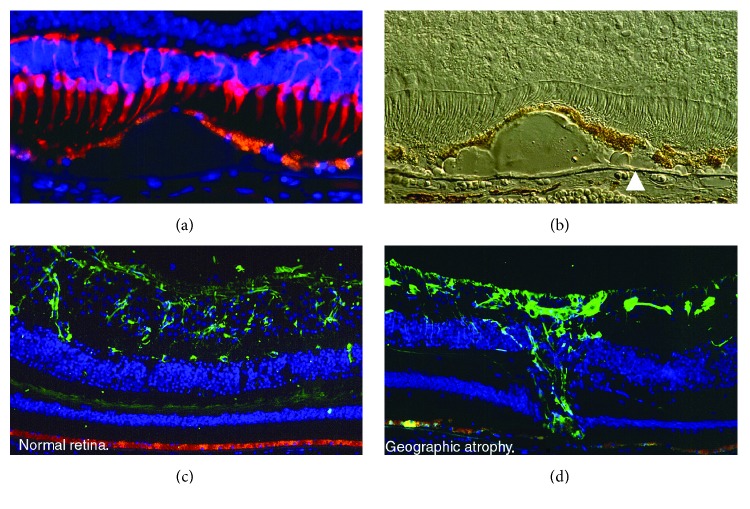
(a) Representative immunofluorescence image of the macula with geographic atrophy and loss of cones (red cells, mAb 7G6) over drusen. The RPE (orange) is thinned over drusen. Cell nuclei are blue (DAPI). 40x objective. (b) Nomarski image of the previous image. Note refractile drusen on Brunch's membrane (arrowhead). 40x objective. (c) Representative immunofluorescence image of the macula in a normal retina. Orange (RPE) and green (GFP) in astrocytes (anti-GFAP). (d) Representative immunofluorescence image of the macula with geographic atrophy. Orange (RPE) and green (GFP) in Müller cell scar (anti-GFAP). Photo credit: “The Human Retina in Health and Disease” Teaching Set by Ann H. Milam Ph.D., University of Pennsylvania.

**Figure 2:**
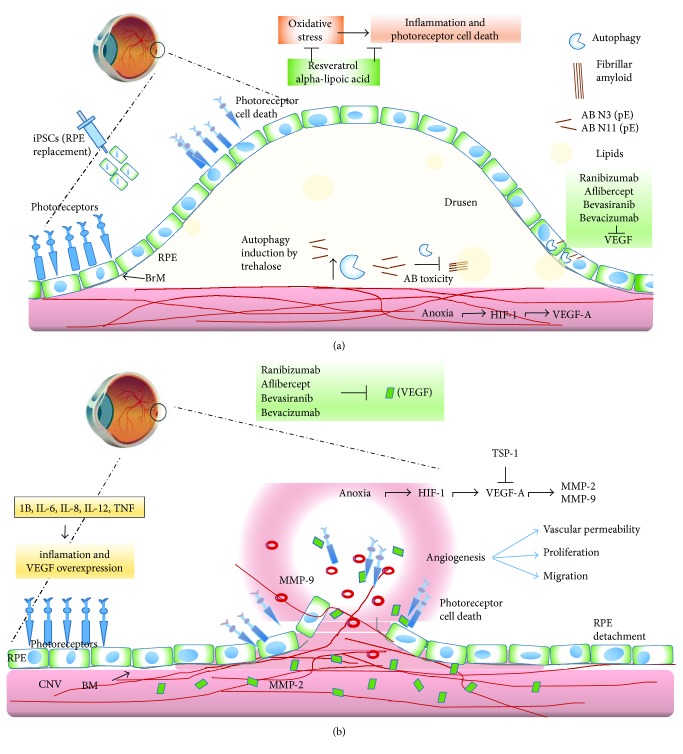
A diagram illustrating the anatomical differences between RPE and BM on dry AMD (a) and wet AMD (b). Early AMD involves the accumulation of drusen and beta-amyloid peptides in the subretinal space. This might progress to dry AMD (a), which is characterized by inflammation and photoreceptor degeneration, caused in part by oxidative stress; resveratrol and alpha-lipoic acid prevent these effects. Autophagy induction by trehalose might help to eliminate intracellular components that abnormally accumulate intracellularly avoiding the following extracellular accumulation of toxic peptides, like beta-amyloid and lipids. Another strategy for the physiological recovery in AMD is the administration of induced pluripotent stem cells (iPSCs). Wet AMD (b) in which neovascularization from invading choroid vessels and the Bruch's membrane (BM) rupture cause photoreceptor damage. Besides, neovascularization of the retina ruptures the Bruch's membrane, which damages the macula and results in blurry or spotty vision. Anoxia and hypoxia-inducible factor 1 (HIF-1) induce the expression of VEGF-A, and as a possible treatment, thrombospondin-1 (TSP-1) protein might be used to block VEGF-A and metalloproteinases 2 and 9 (MMP-2 and MMP-9). Additionally, ranibizumab, aflibercept, bevacizumab, and bevasiranib could be used to block the angiogenic effects of VEGF on both cases.

The hallmark of early AMD is the formation of drusen, pigmentary changes at the macula, and mild to moderate vision loss (Figure 1). There are two major advanced forms of the disease: the “dry” or atrophic form is the most prevalent and is characterized by slow progressive dysfunction of the retinal pigment epithelium (RPE), photoreceptor loss, and retinal degeneration [1, 2] (Figure 2(a)). The “wet” or neovascular form is less frequent but is responsible for 90% of acute blindness due to AMD. It is characterized by choroidal neovascularization (CNV) with intraretinal or subretinal leakage, hemorrhage, and RPE detachments [1, 2] (Figure 2(b)). Both forms are not mutually exclusive. It is known that the dry form can eventually develop CNV and patients with CNV may display some degree of atrophy after a few years [3].

The treatment of the wet form had a major breakthrough due to the introduction of antiangiogenic drugs; suddenly, the functional prognosis changed from almost-certain blindness to more than 90% chance of three-line visual improvement after two years of treatment [1, 4]. Nevertheless, even after this progress, therapy is far from being perfect and there is still ample room for improvement.

There are three main drugs that provide indirect antiangiogenesis by blocking vascular endothelial growth factor (VEGF) in the retina. Ranibizumab (Lucentis, Genentech Inc., South San Francisco CA; commercialized worldwide by Novartis) was approved by the Food and Drug Administration (FDA) in 2006 for the treatment of neovascular AMD [1]. It is a recombinant humanized Immunoglobulin (Ig) G1 kappa isotype monoclonal antigen-binding fragment (Fab) that targets and binds VEGF-A with high affinity. Several clinical trials have proven that a monthly intravitreal dose of 0.5 mg ranibizumab results in the stabilization and improvement of visual acuity in 95% of the patients with neovascular AMD [1]. Aflibercept (Regeneron, Tarrytown, NY; commercialized worldwide by Bayer AG) was approved by the FDA in 2011. It is a fusion protein that combines two key binding domains of human VEGF receptors 1 and 2 and a fragment crystallizable (Fc) region of a human IgG1 [3]. This specially designed protein has a higher affinity for VEGF-A than its natural receptor. In addition, it is capable to bind and effectively block VEGF-B and placental growth factor 1 (PGF1) [1, 3]. Because of its greater half-life, the drug can be used in a bimonthly regimen, which greatly reduces the number of necessary intravitreal injections without losing efficacy [3]. Finally, bevacizumab (Genentech Inc., South San Francisco, CA; commercialized worldwide by Roche) is a full-length humanized antibody that binds and blocks all VEGF isoforms. Despite being an off-label drug for the treatment of AMD, it is the most used ocular antiangiogenic and the 7th best-selling drug in the world with revenues over 6.5 billion US dollars in 2016 [1].

The antiangiogenic treatment is aimed at treating the consequences of long-lasting cellular damage (vascular endothelial growth factor (VEGF) production) but adds nothing to the prevention and prophylaxis of AMD development. In reality, this therapy is not modifying the course of the disease at all but merely resisting its impact through time and delaying its progression. Moreover, there is no proven treatment available for the dry form of AMD. This is in part because until now, the results of research on AMD are just beginning to unravel the intricate relationship between genetic predisposition, environmental factors, and the normal aging process that take place as part of the disease mechanisms. A better understanding of these relationships will help us to identify better potential treatment targets for both wet and dry forms of AMD.

Therefore, the aim of the current manuscript is to review some of the principal molecular mechanisms associated with the pathogenesis of AMD such as the principal mechanism associated with neovascularization through VEGF signaling, oxidative stress, dysregulation of the mechanisms of clearance of proteins and organelles in AMD (autophagy), the pathophysiology of glial cells in the retina on AMD, and closing each subject of this review explaining some of the new potential treatment alternatives for AMD. We have divided the review into two big major subjects: molecular mechanisms and cellular mechanisms, for a better understanding of this work.

## 2. Molecular Mechanisms

### 2.1. Associated Factors with Neovascularization in Age-Related Macular Degeneration

The AMD process starts with atrophic formation, called dry AMD, characterized by decreased vision principally caused by retinal dysfunction, subsequently, developing to a wet condition. The latter occurs when abnormal blood vessels behind the retina start to grow under the macula; these new blood vessels are very fragile and often have blood and fluid leaks [5, 6]. The blood and fluid raise the macula from its normal position in the back of the eye, and the macula is damaged quickly favoring the loss of central vision.

The most studied factor related to ocular neovascularization is the vascular endothelial growth factor (VEGF) [7]. VEGF was first identified as a signal protein of vascular permeability. The VGEF gene encodes a family of glycoproteins generated by alternative splicing, whose primary function is the formation of blood vessels de novo (embryonic development) and angiogenesis (formation of new blood vessels from preexisting vessels) by activating cellular signal pathways.

Members of the VEGF family (VEGF-A, VEGF-B, VEGF-C, VEGF-D, and VEGF-E and the placental growth factor (PGF)) are proteins of approximately 40 kDa. The VEGF-A's biological activity is dependent on proteolytic processes; the products obtained from this degradation interact differentially with VEGF-R1 and VEGF-R2 receptors [8].

Within this group of proteins, it has been reported that the VEGF-A protein induces vascular proliferation and migration of endothelial cells and is essential for both physiological and pathological angiogeneses.

In several diseases, such as rheumatoid arthritis, cardiac ischemia, psoriasis, growth tumor, and diabetic retinopathy, as well as in AMD, the activity of the VEGF-A protein plays an important role. However, the VEGF released in these diseases is due to different factors. The best-studied mechanism of VEGF-A release is associated with the lack of available oxygen; thus, the production of VEGF can be induced in hypoxic cells. When cells are in a low-oxygen microenvironment, they produce the transcription of the hypoxia-inducible factor 1 (HIF-1) inducing the release of VEGF-A (Figure 2(a)). VEGF-A is a heparin-binding homodimeric glycoprotein that acts via endothelial-specific receptor tyrosine kinases, VEGFR1 (Flt1), VEGFR2 (KDR/Flk1), and VEGFR3 (Flt4), located in endothelial cells and in other cell types, and it is known that the most important for angiogenesis is the VEGFR-2 receptor [9].

Once VEGF-A binds to the receptor, several signaling pathways are activated. These pathways are the following: (1) the Mitogen-activated protein kinase- (MAPK-) p38 signaling pathway, where the effector protein (the heat shock protein HSP27) acts by reorganizing actin, (2) the phosphatidylinositol 3-kinase- (PI3K-) AKT protein kinase B pathway, promoting the formation of nitric oxide (ON), and (3) the phospholipase C-gamma (PLC*γ*) triggering the intracellular calcium release, promoting prostaglandin production, and increasing vascular permeability. The three pathways promote angiogenesis [10].

#### 2.1.1. Other Signaling Pathways Involved in Angiogenesis

Currently, other proteins associated with VEGF-A signaling are involved in corneal neovascularization. The best-described proteins related to these processes are metalloproteinases 2 and 9 (MMP-2 and MMP-9). These proteins have acidic properties, are rich in cysteine, and promote angiogenesis as they act to degrade the extracellular matrix, increasing the filtering of molecules that modify the microenvironment finally promoting the formation of new blood vessels [11] (Figure 2(b)).

During the development of AMD, there is a balance between angiogenic and antiangiogenic factors and the loss of this balance favors the development of blood vessels de novo.

Another factor associated with an antiangiogenesis function in AMD is thrombospondin-1 (TSP-1). TSP-1 is a glycoprotein of 450 kDa, a major component of platelet alpha-granules, produced in various cell types such as endothelial cells, monocytes, macrophages, and retinal pigment epithelium (RPE). One function of this glycoprotein in *in vivo* and *in vitro* studies is the inhibition of angiogenesis. In addition, it has been demonstrated that its expression is dependent on the localization. In fact, it is mainly located in the basal lamina of RPE, Bruch's membrane (BM), choriocapillaris, the retinal wall, and the choroidal blood vessels in normal eyes, but in AMD, its expression is significantly decreased, especially in Bruch's membrane and choriocapillaris at the submacular region [12, 13]. These data support the hypothesis that the decrease in the expression and activity of antiangiogenic factors promotes neovascularization. Therefore, we can suggest that the activity of the angiogenic factor VEGF would not be enough to increase vascularization but requires abatement of the activity of antagonist factors.

In another way, the role of cytokines in AMD progression has been controversial. Elevated serum levels of interleukin-6 (IL-6) are associated with an increased incidence and progression of AMD, whereas the presence of interleukin-8 (IL-8) decreases the development of neovascularization and apparently confers a protective effect. However, this topic is not discussed in this review. For a better understanding of inflammation and its role in age-related macular degeneration, authors refer to Kauppinen et al.'s work [14].

#### 2.1.2. Novel Molecular Anti-VEGF Therapies for AMD

Several specific drugs have been developed that inhibit the angiogenic effect or activity of VEGF. In addition to conventional drugs that have been approved so far, currently, progress has been made in the development of small interfering RNAs (siRNAs) as therapeutic agents. These siRNAs are small strands of about 21 nucleotides of RNA that bind specifically to the target mRNA to regulate its expression. Within this group of therapeutic agents, the effect of the siRNA anti-VEGF, named bevasiranib, has been studied [15, 16]. Although, in the clinical trial phases I and II, positive effects in patients were demonstrated, the results in clinical phase III in 2010 were inconclusive and the clinical study was stopped.

A second alternative is the use of siRNA AGN211745 that targets the VEGF receptor 1 (VEGFR-1). The preclinical studies in animal models have shown encouraging results [16]. However, researchers are still working in the development of another specific siRNAs, to provide more advantages in the use of siRNAs and decrease the adverse effects in patients.

#### 2.1.3. Anti-Integrin Therapies

The integrin family of cell adhesion molecules mediates host defense, homeostasis, signal transduction, and various other interactions between the cell and the extracellular matrix. Integrins are type-1 transmembrane glycoproteins expressed on the cell surface widely expressed in choroidal cells and RPE cells and play an important role in the angiogenic pathway.

Three classes of integrin inhibitors are in preclinical or clinical trials: monoclonal antibodies that target the extracellular domain of the integrin heterodimer, synthetic tri-amino acid sequence, arginine-glycine-aspartate (RGD) motif-containing peptides, and peptidomimetics, which are orally bioavailable nonpeptidic molecules that mimic the RGD sequence [17]. A study in a mouse oxygen-induced retinopathy (OIR) model evaluated the in vitro and in vivo pharmacological activity of a novel nonpeptidic integrin alpha v beta 3 (avb3) antagonist, 3-[3-(6-guanidino-1-oxoisoindolin-2-yl) propanamido]-3-(pyridin-3-yl) propanoic acid dihydrochloride (GOPPP), which was shown to inhibit retinal neovascularization. The major results were that GOPPP reduced pathologic but not developmental angiogenesis in neonatal mice. GOPPP effectively reduced pathologic angiogenesis, adhesion, proliferation, and migration, through the inhibition of ERK1/2 and Akt phosphorylation in a model of ischemic retinopathy and its beneficial effects likely involved in the inhibition of retinal VEGF [17].

### 2.2. Oxidative Stress: Implications for AMD

The association between oxidative stress with age-related pathologies, like Alzheimer's disease (AD), Parkinson's disease, atherosclerosis, certain types of cancer, and AMD, is a common finding and has been extensively documented [18–20]. Despite being a disease of unknown etiology, there is strong evidence suggesting that oxidative stress has a major role in the development and progression of AMD [20, 21]. The retina and RPE are extremely susceptible to oxidative stress damage: they both have high metabolic demands and require large amounts of adenosine triphosphate (ATP) to support their functions [22]. The retina has the highest consumption of oxygen per gram of tissue in the human body. Reactive oxygen species (ROS), like hydrogen peroxide, superoxide anions, hydroxyl free radicals, and hydroperoxyl radicals, among others, are readily created as a by-product of increased oxidative phosphorylation in mitochondria [22–24]. In addition, the constant exposure of both structures to UV radiation from white bright light, especially UVA, which is able to excite ocular chromophores and induce DNA damage by secondary photoreactions and indirect photosensitizing reactions, is also a constant source of ROS like hydrogen peroxide (Figure 2(a)) [25–27]. The damage induced by the latter may be enhanced with age due to increased deposits of lipofuscin within the RPE [28]. Moreover, cataract surgery may worsen UV-mediated retinal damage due to the loss of the lens' natural protection, despite implantation of an intraocular lens with a blue-light filter [29].

Environmental insults like cigarette smoking, which is a known inducer of oxidative stress, have been identified as the strongest risk factor for AMD, second only to age (OR 4.5) [26, 30]. When these factors are combined in time, the oxidative burden can build up quickly and eventually surpass the eye's antioxidant capacity. Evidence of this disequilibrium is the finding of many oxidative-modified proteins, lipids, and inflammation-related factors as part of drusen constituents [31]. The dry advanced form of AMD has also been associated with high levels of iron, a prooxidant factor, in RPE and Bruch's membrane [32].

The process of photoreceptors' outer segment shedding and its heterophagy by the RPE is a constant source of polyunsaturated fatty acids like phosphatidylcholine [33]. An environment rich in ROS may induce oxidative modification of excessive phospholipids. In another way, ROS interact with double-bound lipids, inducing their breakdown and giving rise to oxidized forms, like pentosidine, 1-palmitoyl-2-(5′-oxo-valeroyl)-sn-glycero-3-phosphocholine, malondialdehyde, malondialdehyde-acetaldehyde, oxidized phosphocholine, and oxysterols such as 7-ketocholesterol and 25-hydroxycholesterol, among others [33–35]. Most of them can be found as drusen constituents. These newly modified lipoproteins are very reactive and can easily interact with other molecules to form adducts and molecular moieties, which can promote a wide array of effects, mainly the change of nonreactive molecules into epitope-like structures, inducing immune recognition and inflammatory damage via complement cascade activation [34, 36]. This effect can be potentiated further in case of concomitant genetic defects that predispose the patient to the dysregulation of the complement pathway like the H402Y variant or mutations in complement factors H and B [21, 35–37].

Other cellular damages induced by ROS associated with AMD pathogenesis include nuclear and mitochondrial DNA damage, autophagy decline, and induction of programmed cell death of photoreceptors and RPE cells by upregulating the mitogen-activated protein kinase (MAPK), which leads to chronic inflammation and the upregulation of the production of VEGF via ERK1/2 activation [29, 38–42]. They can also act as chemoattractants for systemic macrophages and perpetuate inflammation. Finally, oxidative stress triggers the expression of proinflammatory cytokines, such as IL-1*β*, IL-6, IL-8, IL-12, and TNF-*α*, and depending on which of them is increased, the effect will be either a development or an inhibition of AMD [14]. However, as we mentioned before, we have recommended Kauppinen et al.'s review [14].

#### 2.2.1. Antioxidant Therapies for AMD

In order to restore the balance previously described, antioxidant supplements and ROS scavengers have been proposed as potential therapies for prophylaxis and to decrease AMD's progression (Figure 2(a)).

One of these studies has been entitled “Age-Related Eye Disease Study (AREDS) 1 and 2” and proved, despite their limitations, that nutritional supplementation with antioxidants and micronutrients can effectively reduce the progression toward advanced forms of AMD by 28% over a 5-year period (OR = 0.72; 99% CI = 0.52–0.98), in patients over 55 years of age [43]. It also demonstrated that supplementation of the original formula with lutein, zeaxanthin, and polyunsaturated fatty acids (docosahexaenoic acid (DHA) and eicosapentaenoic acid (EPA)) is safe, although no additional benefit was detected [44, 45].

In another way, resveratrol (3,4,5-trihydroxystilbene) could help for AMD treatment. This molecule is a polyphenolic antioxidant that belongs to the stilbene family, commonly found in grape skin and seeds [29, 46]. It has been recently studied as a potential therapeutic target since it has antioxidant effects against peroxide-induced oxidative stress, reduces the UVA-induced ERK1/2 activation in RPE cells, and reduces MAPK activation and the expression of cyclooxygenase-2 in RPE cells *in vitro* [29, 45–47]. Small case series, using commercially available over-the-counter resveratrol, have shown improvement in retinal structure and function [47]. The use of resveratrol for exudative age-related macular degeneration (AGED) is a phase I/II interventional, prospective, randomized clinical trial (NCT02625376) that will compare the incidence of advanced neovascular AMD between a 250 mg resveratrol bid group and placebo after 24 months of follow-up. The study started in August of 2015 and has a completion date set for 2019. It is currently enrolling, and no results have been released so far.

Another option for AMD treatment is alpha-lipoic acid, which is a cofactor of mitochondrial dehydrogenase. It acts as a free radical scavenger, chelating transition metals, and promotes the regeneration of endogenous antioxidant systems like superoxide dismutase [48]. The use of alpha-lipoic acid in geographic atrophy (GA) is a phase I/II clinical trial, sponsored by the University of Pennsylvania (NCT02613572), which aims to assess the safety and tolerability of 800 mg and 1200 mg alpha-lipoic acid, as well as the change over time in the area of GA in the studied eyes. The study entered phase 2 in May of 2016 and has a completion date set for May 2018. It is currently enrolling, and no results have been released so far.

As we have mentioned before, UV light induces the increase of oxidative damage to the RPE in AMD. The principal associated mechanism to RPE damage has not been clearly described, but oxidative stress has been associated with the overproduction and accumulation of lipofuscin, beta-amyloid peptides, and different proteins, and the accumulation of these molecules is toxic for RPE cells. In AMD, the formation of these aggregates has been related to failures in the normal clearance mechanisms of the cells. One of these processes is called autophagy, and next, we will describe it and its participation in AMD progression.

### 2.3. Autophagy in AMD: Is Cellular Recycling Affected in AMD?

As seen previously in the text, the etiology of AMD is not fully understood yet. Recent information has proposed that failures in autophagy might be a key factor in the development and progression of AMD. We could define autophagy as a normal catabolic process, evolutionarily conserved, that regulates the degradation of dysfunctional cellular and unnecessary components through the formation of a double-vesicle structure, called autophagosome, and its subsequent fusion with lysosomes.

There are three different mechanisms of autophagy: macroautophagy, microautophagy, and chaperone-mediated autophagy. We will focus, here, in an extensive description of this process.

Cellular homeostasis depends on the proteostasis network, and under normal conditions, this can sense and rectify disturbances in the proteome to restore homeostasis in cells. Two of the principal players in proteostasis maintenance are two proteolytic systems, the ubiquitin-proteasome and the autophagy systems. Although there are some differences in these systems, while substrates of the ubiquitin-proteasomal pathway are predominantly short-lived proteins and misfolded or damaged proteins, autophagy substrates are long-lived proteins, damaged organelles, and multiple proteins organized into oligomeric complex or aggregates that cannot be degraded by other systems [49].

In this sense, autophagy has been characterized as a catabolic process that “eats” aberrant organelles, misfolded proteins, and protein aggregates into double-membrane autophagosomes and delivers it to lysosomes [50]. The correct function of this process is important because it is the only known mechanism that eukaryotic cells possess to degrade protein aggregates and the only one by which entire organelles, such as mitochondria and peroxisomes, are recycled [51]. Cell survival is highly dependent on autophagy. In this regard, loss of autophagy particularly causes accumulation of ubiquitin-positive inclusion bodies and triggers degeneration processes [52].

Autophagy is a very complex process and requires a series of coordinated steps. The first step is the formation of an isolation vesicle called phagophore. After the phagophore formation, it elongates around the cytoplasmatic components selected for degradation. The recognition of the components for degradation and the closing of the vesicle are dependent on the lipidated form of LC3 protein (a microtubule-associated protein light chain 3). Then, the lipidated form of LC3 is associated with the outer and the inner membranes of the autophagosome [53, 54]. These autophagosomes are formed by a particular pathway that requires at least twenty proteins called “atg” (autophagy-related proteins) [54]. Finally, the late stage of autophagy (maturation) depends on the fusion of autophagosome and lysosome. This allows contact of autophagosome cargo with lysosomal hydrolases and consequently the degradation of the components that could be recycled (Figure 2(b)). These steps are fundamental for the autophagic flux (the continuous series of events since the cargo is engulfed until it is degraded). Any event that could alter this flux also alters the degradation process and consequently leads to accumulation of autophagosomes. At the end, the cargo degradation is dependent on the interplay between lysosomes and autophagosomes and this is called the autolysosome. In the eye, all cells present autophagy in order to maintain the normal function contributing to healthy vision. These cells express differential autophagy-related proteins, and when there are mutations in these genes, the stress-induced autophagic pathways can be activated inducing the development of ocular diseases [55].

This part of the review summarizes the current knowledge about the role of autophagy in eye health and AMD as potential molecules that could be used as a protective therapy against AMD progression. Many factors activate autophagy in stress conditions similar to those involved in AMD: inflammation, oxidative stress, and hypoxia, and these conditions have been explained before in this review.

Autophagy is especially useful to eliminate or reutilize proteins with a high aggregation propensity. Regarding prone-aggregation proteins or peptides, the beta-amyloid 1–42 peptide (the major toxic peptide observed in Alzheimer's disease) and lipofuscin have been characterized like two of the most prone-aggregation polypeptides in AMD recently. Both polypeptides have gained relevance in AMD because the burden of both increases with age, in RPE-Bruch's region, photoreceptor outer segments (POS), and retinal ganglion cells (RGCs) [56, 57].

We know now that drusen observed in AMD are deposits composed of different intracellular originated proteins and some of them regulate proteolytic processes [1, 58, 59]. In these deposits, the presence of beta-amyloid peptides correlates with age as well as the extent of druse loads observed in AMD [60, 61]. It has been shown that autophagy reduces the toxicity caused by protein aggregates that accumulate in different age-related diseases [58]. Similarly, patients with AMD have shown accumulation of autophagosomes and decreased lysosomes [60, 62].

The presence of such peptides may serve as indicators of an impaired autophagy process in RPE cells that could involve AMD development^,^ [57, 62]. Therefore, preservation of the autophagic activity has been related to a lower intracellular accumulation of damaged proteins, improving RPE cell function and retarding the aging process.

Autophagy induction to clear drusen and beta-amyloid peptides from the macula can be induced by different molecules, and some of them have been proven. It has been reported that autophagy could be involved in the degradation of beta-amyloid peptides through the internalization in clathrin-positive endosomes. However, these mechanisms have not been totally elucidated and current research is being performed in order to probe it in the eye [59, 61, 62].

Among the compounds that induce autophagy, we can find trehalose, metformin, and rapamycin. Trehalose is a disaccharide of glucose, a food constituent produced by different organisms, but it is not present in mammals and it is produced under stress conditions. Its production helps to restore cellular integrity, especially cell membranes. More specifically, in the cornea, this sugar suppresses inflammation and neovascularization. In dry eye, it helps to decrease cell death as well as inflammation. Trehalose has been extensively studied to prevent neurodegenerative disorders, principally by promoting autophagy, reducing the presence of toxic proteins or peptides. Besides, it is not toxic and it could be administrated to humans [63, 64]. In fact, there are trehalose-based eye droops that help to preserve viability and the correct function of cultured human corneal epithelial cells during desiccation [64]. However, its participation in autophagy activation and the mechanisms involved in AMD has not been proven. Another potential use of trehalose is the capability to rescue glial cell dysfunction in mice and to induce autophagy in microglial cells, which degrades beta-amyloid peptides and regulates inflammation in mice [63–65]. For these reasons, we propose that stimulation of autophagy might be a potential therapeutic treatment to decrease the drusen burden, the presence of toxic amyloid peptides, and inflammation. It could be a target for the development of new drugs to retain degeneration processes and prevent AMD development. The pathologies associated with autophagy and AMD are intriguing in their many similarities. Whether one contributes to the other remains to be determined, but now that the reagents are available, experiments can be performed to address this question. This opens up a new area of discovery for AMD.

## 3. Cellular Mechanisms

### 3.1. Pathophysiology of Glial Cells in the Retina and Their Potential as Endogenous Stem Cells

In the central nervous system (CNS), the responses to any pathogenic insult include a prominent participation of glial cells [66]. Glia populations in the CNS consist primarily of microglia, the main resident immune cells, and macroglia, which include astrocytes and oligodendrocytes. These nonneuronal cell populations are intimately integrated into a healthy neuronal function, play important homeostatic roles maintaining the CNS environment, and play a key role in tissue responses to diseases, inflammation, and injury [67–70].

#### 3.1.1. Microglia and Macroglia in the Retina

In the retina, microglia and macroglia are similarly represented. Retinal microglia are found distributed throughout the inner retina in a laminated pattern [71]. Retinal macroglia, consisting of astrocytes and Müller cells (MC), provide support to neuronal functions [72, 73]. As in the CNS and in the retina, both glial cell populations are involved in retinal responses to pathological conditions [74, 75].

While the astrocytic and microglial responses, in an injury context, have been thought to involve cross-talk between these two cell populations, the mechanisms and functional significance underlying these interactions are incompletely understood [76, 77]. MC as well as retinal microglia, similarly, depict marked cellular changes in different retinal pathologies, like AMD [78]. The responses of Müller and microglial cells to injury in the retina have been described as beneficial and deleterious processes [79, 80]. Nevertheless, it is not well known how these types of cells interact in the aftermath of retinal injury and how they shape in adaptive or nonadaptive overall response to the insult.

In healthy retinas, MC cells and microglia are in a constant two-way communication process, where MC signals inform microglia of neural activity and are then integrated to drive a behavioral response in microglia, according to their functions of regulation, synapse modulation, activity, and source of trophic factor release. For instance, neurotransmission between them is a candidate factor for regulating microglial behavior [81, 82]. Current evidence indicates that microglial process motility is sensitive to excitatory and inhibitory forms of neurotransmission. The neural activity induces ATP release, which constitutes the direct signal to regulate the dynamic behavior of microglia [83]. In addition to the release of ATP induced by the activation of metabotropic glutamate receptor [84–86], Müller cells also release ATP by membrane stretch induced by osmotic perturbation as it occurs during neuronal activation [87–89]. In both cases, the ATP release from Müller glia is a Ca^2+^-independent process. This was supported by experimental data which suggest that ATP is released from Müller cells in a SNARE-independent manner, probably via hemichannels [90–94].

Under pathological conditions, microglia react rapidly activating different processes, promoting an activated state in these cells [95, 96]. This microglia condition is the first step of injury response that precedes macroglial responses [97, 98]. The MG responses involve cellular hypertrophy, proliferation, and down- or upregulation of different genes and proteins suggesting that MG respond to microglial activation with an increase in cell-cell contacts and chemokine secretion, which facilitates and guides the radial migration of microglial cell in inflammatory responses in the retina [99]. This Müller glia-microglia response may underlie a mechanism in which an initial detection of injury in a particular locus by microglia may be augmented in magnitude and spatial scale to broaden the adaptive injury response, involving both cell types, to restore homeostasis.

#### 3.1.2. Müller Glia-Like Stem Cells

MC are remarkably resilient to damage and respond to retinal injury and disease by changing their morphology, biochemistry, and physiology [100]. Depending on the severity of the damage, this response may include proliferative events. However, the triggers for proliferative gliosis are not well understood yet. Both proliferative and nonproliferative processes of injury include changes in the gene and protein expression pattern and are often associated with MC hypertrophy.

The nature of the Müller glia is clearly defined by structure, function, and gene expression patterns, providing the neuronal cells with structural, metabolic, and ion homeostasis and synaptic support^.^

However, it should be noted that normal MC have significant transcriptome overlap with retinal progenitors, and there seems to be a gradual transition in phenotype from neural progenitor to mature MC during early postnatal retinal development [101, 102]. Nevertheless, MC should not be referred to as stem cells, given that these glial cells do not function as stem cells in the retina under physiological conditions. Furthermore, MC have also been characterized as the “radial glia” of the retina based, at least in part, on their morphology and radially oriented processes that span the retina from outer to inner limiting membranes. Radial glia in the developing brain has been shown to function as a progenitor and to provide structural guides for the functions of migrating and differentiating neurons that MG do not provide during development, but can provide in a regenerating retina [103].

In the last two decades, MC cells have been considered a source of stem cells of retinal regeneration in fish, chicks, and rodents. The neurogenic potential of MC was first identified in a chicken retina [104] and thereafter in a rodent retina [105]. There is also evidence that MC from the primate retina can become progenitor-like cells in vitro [105] but the potential of these glial cells to regenerate neurons in an intact primate retina remains unexplored. In addition, mammalian MC can respond to injury, proliferate, and express genes associated with retinal stem cells but they do not function as retinal progenitors *in vivo* [106, 107]. Nonetheless, these characteristics suggest that, under the right conditions, MC might be induced to adopt the characteristics of a retinal progenitor that could be used for retinal neuron repair. Indeed, MC cell culture from humans has the capacity to generate both neurons and glial cells [108, 109] suggesting that human MC are capable of generating neurons under appropriate conditions and they could be able to participate in retinal repair and could be used for AMD treatment.

Experimental data showed the expression of neurogenic genes, such as Notch and Wnt, in MG culture that induce photoreceptor progenitors [110]. On the other hand, activation of FGF, Notch, Wnt, and Sonic-hedgehog signaling events induces a significant number of MC cells to reenter the cell cycle and display properties of retinal progenitors in the injured mammalian retina [111–115]. These results indicate that some part of the regenerative cellular program may be induced for retinal repair in patients with retinal degeneration, suggesting that overexpression of Achaete-Scute complex-like 1 (Ascl1) in MC culture induces a neurogenic state of MC, proliferation, and bipolar neuron generation. This has led to propose Ascl1 as a potential target for neurodegenerative therapy after disease or injury [116].

Apparently, microglia lack the neurogenic capacity observed in macroglia stimulated *in vitro* [117]. Nevertheless, it seems that activation of microglial reactivity is an important step in stimulating MC to dedifferentiate, proliferate, and become progenitor-like. It is likely that reactive microglia provide signals to modulate the reentry into the cell cycle [118]. Alternatively, the reactive microglia could suppress inhibitory signals that prevent the formation of MG progenitor-like cells [119]. The identity of the signals provided by reactive microglia to stimulate the formation of progenitor-like MG remains uncertain, but the participation of proinflammatory cytokines and components of the complement system is possible [118]. Another important factor for the neurogenic potential of MG might be the age of the organisms. Experimental studies have shown that MG from the mouse retina ex vivo express neurogenic factors and generate progeny expressing neuronal and glial markers in response to growth factor stimulation; nevertheless, the potential regenerative capacity of MG becomes limited with increasing mouse age [120].

In conclusion, the concept that the adult mammalian central nervous system contains populations of resident neural stem/progenitor cells was accepted two decades ago. Emerging evidence suggests that MG are dormant stem-like cells found throughout the retina and serve as a source of progenitor cells to regenerate retinal neurons after injury, although barriers to regenerative cell survival, migration, integration, and safety concerns remain to overcome. Endogenous retinal repair is progressing rapidly, and the turn of the endogenous stem cells approach into viable therapy might be soon.

### 3.2. Cell Transplantation for Dry AMD, Functional RPE Cells, and Stem Cells

In the past decades, stem cell-based research has become a very promising area in biology. It has been acknowledged that terminally differentiated cells can be successfully reprogrammed [121, 122]. Furthermore, both systemic and local stem cell-based therapies have been used in various diseases with positive results.

While the wet AMD could be treated and fairly controlled by the use of drugs that target the VEGF receptor, the application of laser photocoagulation, and vitrectomy, among other surgical procedures, the dry AMD commonly demonstrates poor outcomes with conventional therapeutic approaches. Damage in dry AMD is mostly attributed to the accumulation of reactive oxygen species and peroxide, in addition to chronic inflammation in the retina that leads to apoptosis of the retinal pigment epithelial (RPE) cells, which gradually damage the photoreceptors [123, 124]. At the present, no treatment can reverse dry AMD; therefore, RPE replacement and retinal microenvironmental regulation represent potential new approaches for dry AMD (Figure 2(a)). [124, 125].

RPE cells can be divided into stem cell-derived RPE cells, fetal or adult RPE cells, iris pigment epithelial cells, and autologous RPE cells [126]. Autologous RPE transplantation as an alternative surgical approach has been extensively studied, generally performed by collecting the healthy RPE in the peripheral retina and transplanting them into the subretinal space at the diseased macula [125, 126]. Fully functional RPE cells can be generated from stem cells or somatic cells by spontaneous differentiation or cell reprogramming [125]. Moreover, RPE cells can be differentiated from human embryonic stem cells (hESCs) or human induced pluripotent stem cells (hiPSCs) [127, 128]. Both the hESC- and the iPSC-derived RPE cells display RPE-like morphology, express typical RPE markers, and have the ability to phagocytose photoreceptor segments [129].

Takahashi and Yamanaka and Yu et al. recently described the iPSCs, which consist of a line of cells reprogrammed by the use of Thomson factors or Yamanaka factors, showing morphological characteristics and differentiation abilities similar to those of the hESCs [130, 131].

In addition, in a study by Carr et al., human RPE cells could be generated from iPSCs by spontaneous differentiation or directed differentiation, as described by Kokkinaki et al. and Kamao et al. in their respective reports [129, 132, 133]. Similarly, Vaajasaari et al. successfully differentiated RPE-like cells from several human pluripotent stem cell lines without the use of animal cells or serum during the differentiation, demonstrating that the appearance of the first pigmented cells was relatively fast, both in hESC and hiPSC lines, varying from 10 to 21 days [134].

Transplantation of intact primary RPE cells has been previously attempted for the treatment of AMD. However, there are several advantages to the use of progeny obtained from hESCs as a source of replacement tissue for clinical studies (Figure 2(a)) [123]. These include *in vitro* differentiation that can be controlled to ensure optimum safety, purity, and potency before transplantation into the selected population of patients [123, 134].

Based on the preclinical data reported, the initial clinical trials, evaluating the performance of the transplantation of hESC-derived RPE cells to subretinal space, were phase I trials designed to test the safety and tolerability of grafted hESC-derived RPE cells in patients with either dry AMD or Stargardt's macular dystrophy [135]. The first data obtained from two of these clinical trials reported no signs of rejection, evident hyperproliferation, or tumorigenesis [136]. Moreover, Schwartz et al. [124] reported recently that within the confines of these phase 1 trials, the transplanted hESC-RPE cells appear to be well tolerated, without the presence of adverse intraocular or systemic events related to the cells [124]. There are, at this time, two other clinical trials using hESC-RPE cells, and both are designed to evaluate safety and tolerability of the injection and/or transplantation of MA09-RPE cells in the subretinal space of patients with dry AMD, recruiting patients aged 55 years and older, who will be receiving between 50,000 and 200,000 MA09-hRPE cells [137].

Conversely, stem cell generation may present challenges as well. Abnormal gene expression has been reported in some iPSCs, in which the T-cell-mediated immune response can be elicited even in syngeneic hosts [138]. In addition, another pending challenge would be the immunosenescence, a process that results in the progressive decline of the fine control in the immune system, including the loss of the CD28 receptor, increased interleukin-17 production, and an increase in the IL-6 receptor. All of these changes could not only annul the immune privilege but could also create an environment appropriate for cell death [139, 140].

Stem cell-based therapies have been the object of an extensive research, and great advances have recently been made towards the generation of stem cell transplantation techniques for the functional replacement of RPE cells and photoreceptors. However, it is crucial to assess the long-term safety and efficacy of the current hESC- or iPSC-based RPE transplantation approach in human patients. Adverse events related to the surgery have to be further studied, and large numbers of patients with microperimetry assessments in conjunction with optical coherence tomography (OCT) and autofluorescence estimation should be evaluated to provide more rigorous structure-function correlations.

Recent promising developments in the functional replacement of retinal RPE cells give rise to the expectation that clinical replacement of damaged retinal cells may be able to improve the outcomes of patients with retinal degenerative disease in the near future.

## 4. Conclusions

As we have seen, there are many factors that influence the origin and progression of AMD and the more relevant pathways associated with chronic retinal degeneration. This opens new windows to provide multiple therapeutic targets for disease treatment. However, the design of new treatments must be very carefully done because most of the altered pathways in AMD are broadly redundant and may induce negative effects. Therefore, the new treatments should be carefully designed to cover different altered mechanisms at the same time. Looking towards the future of AMD therapy, there is an emerging paradigm that diverges from the normal approach of preventing retinal dysfunction and death. We should try to recover retinal health in spite of injury, rather than avoid and eliminate numerous overlapping insults. This kind of research should not be discarded in order to improve AMD prevention and treatment. Some experimental ideas are already being performed in our laboratory.

Finally, it is totally necessary to consider the financial burden that AMD represents due to its progressive nature, for example, loss of productivity, and related expenses like nursing homes, caretakers, and comorbidities, and currently available treatment is a significant challenge to any health system. Currently, it represents an excessive direct annual medical cost and, as the incidence raises with the passing of time, the annual cost will increase; for this reason, the finding of a new functional treatment for AMD becomes totally needful.
